# Melanin and Melanin-Related Polymers as Materials with Biomedical and Biotechnological Applications—Cuttlefish Ink and Mussel Foot Proteins as Inspired Biomolecules

**DOI:** 10.3390/ijms18071561

**Published:** 2017-07-18

**Authors:** Francisco Solano

**Affiliations:** Department of Biochemistry and Molecular Biology B and Immunology, School of Medicine and LAIB-IMIB, University of Murcia, 30100 Murcia, Spain; psolano@um.es; Tel.: +34-868-887194

**Keywords:** melanin, polydopamine, adhesive material, biofilms, coated nanoparticles, therapeutical devices, biomedical applications

## Abstract

The huge development of bioengineering during the last years has boosted the search for new bioinspired materials, with tunable chemical, mechanical, and optoelectronic properties for the design of semiconductors, batteries, biosensors, imaging and therapy probes, adhesive hydrogels, tissue restoration, photoprotectors, etc. These new materials should complement or replace metallic or organic polymers that cause cytotoxicity and some adverse health effects. One of the most interesting biomaterials is melanin and synthetic melanin-related molecules. Melanin has a controversial molecular structure, dependent on the conditions of polymerization, and therefore tunable. It is found in animal hair and skin, although one of the common sources is cuttlefish (*Sepia officinalis*) ink. On the other hand, mussels synthesize adhesive proteins to anchor these marine animals to wet surfaces. Both melanin and mussel foot proteins contain a high number of catecholic residues, and their properties are related to these groups. Dopamine (DA) can easily polymerize to get polydopamine melanin (PDAM), that somehow shares properties with melanin and mussel proteins. Furthermore, PDAM can easily be conjugated with other components. This review accounts for the main aspects of melanin, as well as DA-based melanin-like materials, related to their biomedical and biotechnological applications.

## 1. Introduction. Melanin as a Relevant Biomaterial

Natural melanin is a structurally ill-defined polyphenolic material widely found in all types of living organisms, from bacteria to mammals. In animals, melanin is found in the skin as well as hair, and some neurosensorial tissues, like cochlea, retina, and substantia nigra. Melanin is heterogeneous, but it can be roughly classified into two large types, dark eumelanin, and red to yellowish pheomelanin [1,2]. The main difference between these two types resides on the absence (eu) or the presence (pheo) of sulfur in the melanin composition. In pheomelanin, sulfur comes from the conjugation of the amino acid cysteine, or some of its derivatives, such as glutathione, with phenolic precursors. In plants and fungi, other melanins can be found, generally called allomelanin. They are basically devoid of nitrogen and sulfur, as formed by polymerization of different catecholic and dihydroxynaphthalene precursors [2].

In fact, catechols (ortho-dihydroxyaryl compounds) are biomolecules involved in many biochemical processes and functions. Their occurrence in not limited to melanins. Catechols are easily oxidized to semiquinones and o-quinones, and can give rise to cross-linked polymers that are considered o-dihydroxyaryl macromolecules [3]. These polymers are either naturally-occurring in living cells, or chemically synthesized. They easily react with several chemical groups to establish reversible equilibria of moderate redox potential with versatile physical, mechanical, and chelating properties. Catechols and their related o-quinones react with nucleophilic amine and thiol groups, either by means of the Schiff base reaction, or alternatively, through the Michael addition reactions [4]. These reactions also give rise to aryl–aryl covalent linkages among units, or conjugated adducts with other agents. In that way, l-dopa, DA and other catechols, either as monomers or as catechol-containing polypeptide and polysaccharide chains, are able to form a variety of protomolecules with interesting applications in material science and bioengineering.

During the last years, it a growing interest has developed for “green” (bio)electronics. This is the identification of new biomaterials that can be used for the construction and design of biodegradable and biocompatible immune-tolerant devices [5,6]. Melanin is a biopolymer derived from natural sources, so that it shows good biocompatibility and biostability. The first term can be defined as the absence of side effects, including cytotoxicity and antigenic response, when it is added to cell culture [7,8,9] or injected to living organisms [10], and the second, as the relative long life due to the absence of specific melanin-degrading enzymes in living cells, On the other hand, melanin shows a series of useful physicochemical properties, including a broadband absorption for UV–Vis–infrared radiations, hybrid ionic–electronic conductance, metal chelation, free radical scavenging capacities, redox reversibility [11,12,13], easy and cheap availability, and chemical versatility by conjugation with other molecules. In this context, natural and synthetic melanin-like molecules are excellent candidates with unique properties for biotechnological applications [14,15].

### 1.1. Melanin (Eumelanin) Structures

For decades, natural eumelanins have been considered heterogeneous polymers formed by aryl–aryl covalent bonds among cycled and uncycled dopa and their derivatives. Briefly, in the biosynthetic route, or Raper–Mason pathway, l-tyrosine/l-dopa are oxidized to l-dopaquinone. Then, either thiol-conjugation or cyclation of that o-quinone to l-dopachrome gives place to pheo- or eumelanins. In the last kind, subsequent formation of dihydroxyindoles and polymerization leads to eumelanin polymer [1,2,16]. Although this polymer was considered structurally undefined, the degree of polymerization of eumelanin had been widely studied [17,18], but it had remained elusive to a number of chemical and spectroscopic analysis methods, due to the insolubility and untreatability of the final pigment.

**Figure 1:**
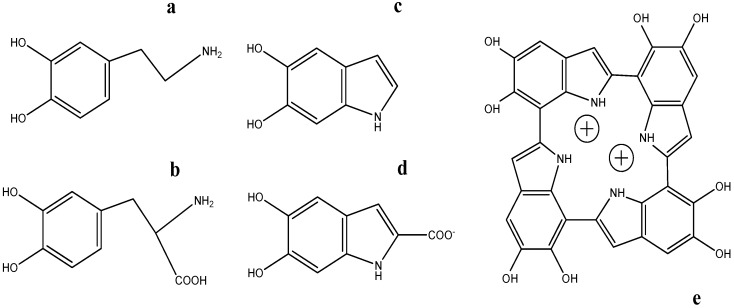
Structural units of natural eumelanin and polydopamine melanin (PDAM). Monomers (**a**) dopamine (DA); and (**b**) Dopa, DHI; (**c**) DHICA; (**d**) the porphyrin-like tetramer; and (**e**) protomolecule proposed as a putative symmetrical unit of melanin polymer to account for some properties, such as the high voltage output of battery devices built with PDAM coating on graphene or metal oxides. This tetramer can be a constituent of both natural melanin and synthesized PDAM.

Melanin properties are more studied and defined in eumelanin than in pheomelanin. Natural eumelanin is formed by oxidative polymerization of l-dopa (Figure 1b) through mixed reactions of carboxylated and decarboxylated intermediates, mainly DHI, DHICA (Figure 1c,d), as well as low content in uncycled dopa units and carboxylated pyrroles formed from the cleavage of dihydroxyindoles during oxidative reactions. Carboxylated and decarboxylated dihydroxyindoles are formed after dopa reductive cyclation by a Michael addition of the side-chain amino group [1,2,16,19]. The final product seems to consist of a certain unordered structure, with undefined and tunable effects on the corresponding optoelectronic properties [20].

Furthermore, recent studies have shown that slight variations in the monomer structure of melanin introduce significant differences in its antioxidant and redox features [21]. These variations are primarily governed by the extent of decarboxylation of l-dopachrome at the critical branch point of the eumelanin pathway [2,16]. Thus, the DHICA/DHI ratio of natural eumelanin affects the overall properties of the resulting pigment, especially its redox and metal-chelating activity, but it also affects the morphology of the natural melanin granule.

The development of novel methods for studying condensed-matter physics and chemistry of materials, including imaging techniques and theoretical calculations, has allowed the improvement of melanin and its supramolecular structure. Hence, new data obtained in the last years point out that melanin is not merely a highly polymerized network as has been believed. The study of broadband UV–Vis absorption spectra of eumelanin, and the associated optoelectronic properties of this material, have been considered a cornerstone for elucidation of its molecular structure [20,22]. The absorption profile is indeed consistent with an amorphous and disordered polymeric semiconductor, but the UV–Vis spectra can be alternatively explained by the chemical disorder model. This model proposes that natural eumelanin consists of many chemically distinct protomolecules. The broadband absorption would be the average of the different absorptions of every species in the UV–Vis range. The chemical disorder model was later complemented with the geometric disorder model to account for the excitonic interactions among melanin protomolecules [23,24]. Furthermore, the absorption spectra profile is not constant during the eumelanin formation, and its evolution may reveal some temporal reorganization of the polymer [25] that might be related to the cross-linking linkages between aryl units after melanin formation or light exposure, making aged melanin molecules bigger and really polymerized within covalent binding network [2,26].

Thus, fresh eumelanin seems to be formed by a rearrangement of protomolecules. The size of these protomolecules is compatible with trimers, tetramers, pentamers, and octamers of dopa-derived dihydroxyindole units. These protomolecules arrange with a defined interlayer distance, forming onion-like nanostructures. The oligomers are mainly formed by 2→4′, 2→7′, or 4→7′ bi-indole cross-linked covalent bonds among units [16]. Due to its symmetrical structure, special interest is devoted the proposed porphyrin-like tetramer structure [22,27]. This arrangement of the four-membered ring protomolecule (Figure 1e) has the correct number of exposed nitrogen atoms in the core to bind ions and create larger molecules with high potential energy storage properties usable in alternative batteries [28].

As an attempt to simplify the structure of melanin and its interpretation, DA (Figure 1a) was introduced instead of l-dopa, to synthesize pigment totally devoid of carboxylic groups. Using DA as precursor, this catechol is oxidized to dopaminequinone, and DHI units without DHICA in the final pigment. PDAM was first used in many studies looking for the synthesis of a more reliable melanin that can be used as material in electronics [29,30]. Sometimes, instead of DA, oxidative polymerization of preformed DHI has also been a model for melanin synthesis. In order to get more treatable soluble pigment in colloidal solutions, modifications of this unit had also been introduced, such as 5,6-dihydroxy-3-indolyl-1-thio-β-d-galactopyranoside [31], or DHI polymerization in the presence of 1% of polyvinyl alcohol [32,33].

**Figure 2:**
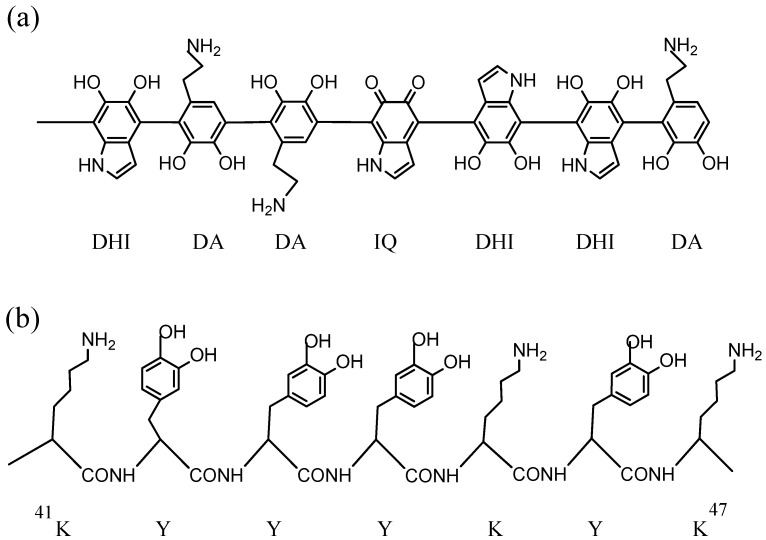
(a) Proposed PDAM linear chain fragment. Units are linked by 4→7′ bonds. Uncycled and unordered DA, DHI, and 5,6-indolequinone units are represented; (b) Peptidic fragment (positions 41 to 47) of mfp5, ^41^KYYYKYK^47^. Side chains of catechol (after tyrosine hydroxylation) and lysine residues are drawn. Hydroxy and amine groups are represented undissociated. The number of possible oxidation of catechol residues to quinones is unknown in both molecules, but cycling to indole units is only possible in PDAM. As higher the number of oxidized o-quinone residues, as lower the adhesive properties of mfps.

The analysis of the DPAM polymer formed with DA showed some differences with previously proposed models for melanin. PDAM could consist of a supramolecular aggregate of monomers held together through a combination of charge transfer, π-stacking, and hydrogen bonding interactions, rather than oligomers consisting of covalent linkages among units [34]. The π-stacked unit without covalent bonds could be DA units [34], but it was extended to DHI [35]. Opposite to that, it was also published that PDAM are chains of oligomers containing DA monomer, cycled DHI, and o-quinone units covalently linked by C–C bonds between aryl rings (Figure 2a), disposed in a parallel or antiparallel manner [36].

In summary, according to recent studies on energy absorption, structural analysis and theoretical interpretations, several somewhat contradictory hypotheses have been published. In fact, the issue of the elucidation of the structure of eumelanin, or even of the simplified PDAM, is really a never-ending story [36]. It is very likely that the structure of PDAM and any melanin could depend on the conditions of oxidation and polymerization [37].

### 1.2. The Relationship between Melanin, PDAM and Adhesive Materials

In the previous section, DA has been introduced as a decarboxylated catechol precursor that simplifies eumelanin formation in comparison to l-dopa, and the “in vivo” precursor of natural melanin after tyrosinase-catalyzed o-hydroxylation of l-tyrosine. However, in a different line of research, other melanin-related catecholic biomaterials with unique properties have drawn great attention in bioengineering research. Mussels are marine fouling organisms able to anchor to wet surfaces by formation of an adhesive resistant material that has bioinspired its use in applied sciences [38,39]. The robust adhesive properties are due to the synthesis, in the mussel byssus, of up to six small foot proteins (termed mfps). These proteins contain in their sequences, a high number of basic residues, and more importantly, of catechol as side chain residues (derived from tyrosine post-translational modification) that are responsible for the adhesive plaque. Mfp5 initially contains 30% tyrosine residues, and mfp3, 26% [39]. These catechol residues resemble dopa/dopamine units integrated in a protein chain by peptide bonds. Figure 2b shows a fragment of mfp5 (^41^KYYYKYK^47^), but others are similar, such as the fragment ^4^YYPKYKY^10^ in mfp3. The amount of mfps in mussel is too small to allow natural extraction, but their surface-adhesive properties have bioinspired the synthesis of mimicked catecholic chains for the development of adhesive and self-healing materials.

**Figure 3:**
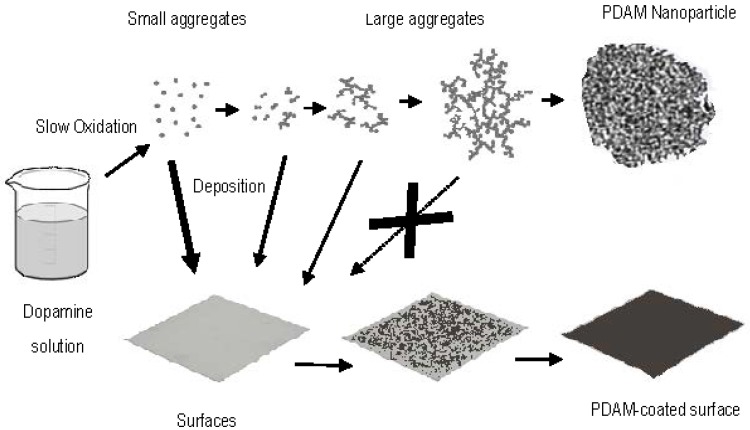
DA oxidation and formation of bare PDAM nanoparticles or deposition of a PDAM film on a surface of diverse material. At the beginning of the oxidation, DA is forming a stable colloidal suspension that is aggregating to particles of growing size, eventually into spherical PDAM nanoparticles. DA monomers and very small aggregates show adhesive properties, and they are able to deposit on a surface for coating, but after a threshold size, large PDAM nanoparticles do not coat, just precipitate.

Taking into account the presence of catechol and basic amino groups in mfps, DA was the immediately bioinspired molecule, as it contains both chemical groups, and thus presumably has the capacity to form adhesive films. DA indeed polymerizes to PDAM in a single step in moderate basic aqueous solutions [40,41], or hydroethanolic solutions with low concentrations of Tris base, ammonia, sodium hydroxide, and other basic catalysts [42]. Pure DA solutions give place to black PDAM nanoparticles that are considered eumelanin-like granules. The size of these nanoparticles is similar to those of natural melanin granules in eye choroid or sepia ink [43]. More interesting, DA oxidation is able to deposit PDAM on surfaces of organic or inorganic materials immersed in the solution, forming an adhesive film coat (Figure 3). In principle, the thickness of PDAM coating ranged from tens to hundreds of nanometers, depending on conditions. For instance, increasing the pH of the DA solution from 8.2 to 10 dissociates hydroxyl groups in the catechol units, and inhibits PDAM adsorption on negatively charged surfaces.

PDAM synthesized from DA solutions shows different adhesive features than natural mfps in mussel adhesive plaques, due to several reasons. Firstly, DA can cycle to indole units during oxidation to PDAM, but the catecholic side chains in mfps cannot. Secondly, the spatial disposition and redox potential of the catechol and basic residues in both types of molecules is similar, but not coincident. PDAM shows chains of consecutive catechol-related units, but mfps show abundant catechols separated by other amino acid units, according to the protein sequence (Figure 2b). The reactivity and arrangement of the catecholic residues is surely affected by the vicinal residues. In fact, the adhesive properties of the six mfps differ due to their different sequences [39]. Thirdly, the basic amino groups in mfps should contribute to the adhesive force. Similar adhesive material to mfps, but with higher adjustable mechanical properties, have been found in the jaws of the sea worm *Nereis virens* [44]. This is due to the protein Nvjp-1, structurally not related with DA or melanin, as it contains more than 25% of histidine, but not tyrosine-derived, catechol residues. In that case, the adhesive mechanical properties are due to the quelation of metal ions by the H.

During DA oxidation, PDAM is able to be deposited on the surface of any material, including ceramics, metals, metal oxides, and hydrophilic and hydrophobic organic polymers, even anti-adherent PTFE. The coating films are robust, and they show wide utility by themselves, or for ulterior conjugation with other biomolecules, to get nanoparticles showing modified sizes and properties, due to the outer second surface-covering [45]. Thus, DA and the resulting PDAM is currently one of the preferred materials to get nanoparticles for biomedical applications [29,30].

Although the details on DA polymerization and final structure of the PDAM depend on the conditions of the synthesis, it is likely to involve the oxidation of catechol to o-quinone and cross-linkages among units. Bearing in mind the adhesive properties of mfps described above, it is well known that the o-hydroxyl groups of catechol show strong noncovalent interactions with surfaces of different nature, meaning strong adhesive forces, while the oxidized o-quinones do not [39,46]. As a result of lower degree of oxidation and o-quinone formation, there is better adhesion but presumably a lower degree of PDAM cross-linking among units.

In an attempt to fuse melanin and mfps, melanin-like short peptides can be easily prepared, and the position of the catechol residues can be appropriately chosen for getting new materials and exploring the effect of specific amino acids in the structure of melanin-related peptides. Recently, Hekstra et al. [47] synthesized some tyrosine-containing tripeptides, in combination with phenylalanine and aspartic acid as the other two components. After tyrosinase oxidation, these tripeptides form melanin-like pigments, but the spatial arrangements in the generated polymers remarkably depend on the position of the catecholic residue, especially on the positions of aspartic acid. This seems to be due to covalent and non-covalent disorder factors that give place to several structural arrangements over a considerable range. The functionality of these melanin-like peptides can be tuned, as well as their morphology, light absorption, and electrochemical properties. Thus, synthetic tyrosine-containing oligopeptides open up a new way of tuning melanin-bioinspired molecules after activation with tyrosinase, for the addition of catechol groups.

### 1.3. Natural Melanin versus Synthetic DPAM

The comparison of natural melanins versus synthetic PDAM has been eventually studied in relation with the electrical and optoelectronic properties [15,37,48]. It is clear that natural melanin granules extracted from sepia ink, hair, choroid, and other sources are finished materials with spherical or ovoidal shapes, comparable to nanoparticles, but with very limited adhesive properties. On the other hand, PDAM is formed “in situ”, and it is much more convenient for the formation of nascent nanoparticles or coating. PDAM can be deposited on different surfaces for coating (Figure 3), or co-assembled with organic monomers to get composite nanoparticles. In the last years, synthetic PDAM has displayed natural melanin granules in most bioelectronic applications, and DA is preferred to l-dopa to get adhesive films and tailored melanin-like material.

On the other hand, looking for differences between natural granules and synthetic PDAM nanoparticles, melanin from cuttlefish ink or animal hair is formed by tyrosinase-catalyzed hydroxylation of tyrosine (tyrosine hydroxylase activity), and subsequent dopa oxidation (by dopa oxidase). This process is regulated by the amount of enzyme, rather than the chemical reaction of melanogenic intermediates [2]. However, DA chemical oxidation displays faster reaction rates at basic pH. Furthermore, natural melanin is formed inside melanosomes, with the presence of small amounts of proteins and other biomolecules.

There are some reports on biotechnological applications of natural melanin granules [14,20,48] that suggest that natural melanin behaves more efficiently than synthetic melanin. For instance, natural melanin from sepia ink seems to exhibit higher specific capacity than synthetic melanin in aqueous sodium-ion batteries used as energy storage devices [49]. Although the molecular reasons are unknown, the higher efficiency would be related to the unique nanostructure of those melanin granules from the ink sac. More recent studies have reported that natural melanin might be a powerful material for use as a cathode of batteries, in sodium ion batteries with sodium titanium phosphate as anode [28]. The voltage output is high enough to be considered an alternative for lithium ion batteries. The energy-storage pattern could indicate that melanin has the porphyrin-like tetramer structure, and sodium ions occupy the core of those tetramers with indole nitrogen atoms (Figure 1e) [22,27]. However, synthesized PDAM is also consistent with the tetramer model, so that the unique pattern of natural melanin, if so, would be at least partially related to other features that can be globally related to the electret state [50]. This is dielectric material that contains electric charge, and an almost permanent dipolar polarization. Natural eumelanin shows a high electret state, which is important to improve the optoelectronic properties of materials, as it allows the generation of permanent and inducible internal and external electric fields. To this regard, the electret state depends on the hydration degree of the molecules, and the presence of small amounts of proteins in the structure [50,51]. Natural melanin from sepia ink and animal choroid contains more water and protein than synthesized PDAM.

### 1.4. Effect of Media Conditions on DPAM Formation

Conditions for the preparation of melanin-like films on several kinds of materials have been carefully studied at different conditions of pH, buffers, and oxidants [40,42,52]. PDAM coating can occur in a single step, but this process is not as simple and reliable as sometimes claimed. Specialized literature prove that the oxidation conditions remarkably affect the aggregation of DA to get colloidal nanoparticles, or to deposit on surfaces of differing nature.

Presumably, synthetic PDAM is usually obtained from DA by oxidative polymerization. Hong et al. [53] revealed the existence of self-assembled trimers of DA_2_/DHI, pointing out that PDAM contains uncycled DA and cycled DHI. The initial DA concentration changes remarkably in terms of the relative proportions of the cycled and uncycled units, the composition of oligomers, and the adhesive properties of the film. Variable contents of three components (DA, DHI and a few pyrrole-carboxylated units), were detected in the structure of PDAM [54].

The preparation of PDAM from other precursors differing from DA, such as previous cycled DHI [32,33], obviously excludes the presence of DA units. Melanin from DHI has been formed using peroxidase/hydrogen peroxide in phosphate buffer, pH = 7 [31,32], and this system introduces differences in comparison to oxidation by air in basic media, making PDAM more similar to natural melanin.

Aside the precursor nature, pH, buffer, oxidant, and the nature of the surface to be coated, also affect PDAM formation and properties.

The deposition rate of precursor is pH-dependent. DA oxidation by oxygen in diluted sodium hydroxide, ammonia, or Tris buffer at pH around 8.5, yields an insoluble black film/powder in a few hours [55,56]. Ammonia is preferred in ethanol/water mixtures, for an easier rinse and dry. The growth rate of PDAM film is maximal at approximately pH 9.5, but is almost completely abolished at higher than pH 10, because of increased DA solubility [56].

The increase in the ammonia concentration decreases the diameter of the particles. At pH around 9, DA solutions give place to spherical nanoparticles that are smaller than natural melanin granules from *Sepia officinalis*, and more uniform in diameter, composition, structure, and durability [22,43]. This might be beneficial for some purposes, as formation of PDAM nanoparticles used for body inoculation, since small size minimizes the recognition by phagocytes.

The use of Cu(II) as oxidant, instead of atmospheric O_2_, modifies the broadband absorption spectra, and increases the thicknesses of the films [52]. Recently, it has been reported that Cu(II)/H_2_O_2_ can trigger the rapid oxidation of DA for a very homogeneous, highly uniform, smooth and stable PDAM coating, proposing that this oxidant is better in comparison to slow or very quick DA oxidation with air or with strong oxidants, as (NH_4_)_2_S_2_O_8_ [56].

DA can also polymerize to PDAM in the absence of oxygen, and in acid media using plasma-activated water [57], or electrochemical deposition using cycling voltammetry [58]. These methods are more sophisticated that DA oxidation in air, but they seem to allow a better control of the film thickness, as well as more stable PDAM.

Although PDAM is deposited on virtually any surface immersed in a solution of DA, the chemical nature of the surface also affects the nucleation and growth of the PDAM film. For instance, the density and surface coating on SiO_2_ is improved when the pristine silica is first covered with monolayers of neutral aromatic or aliphatic substrates [59]. A monolayer of octadecyl is more effective than aminopropyl, suggesting that hydrophobicity increases the nucleation density, rather than reactions with extra amino groups.

### 1.5. Types of PDAM-Containing Nanoparticles. PDAM Combination with Other Materials

Oxidation of DA solutions gives place to a slow darkening, followed by formation of an unstable colloidal solution formed by very small microscopic particles that evolve to large aggregates around 50 nm diameter. Subsequently, precipitation of a black powder occurs, consisting of roughly spherical PDAM nanoparticles.

The aggregation of DA proceeds by two processes important for coating different surfaces. Small nanoaggregates until around 50 nm can deposit onto de growing film, but larger ones tend to precipitate, with poor adhesive properties. Very small particles, and DA oligomers formed in suspension, can be incorporated to the film, but once they reach a threshold size, they cannot, and precipitate (Figure 3) [40,52,56]. In consequence, PDAM coating is more uniform and robust when the DA solution is refreshed, as incorporation of new DA monomers is more efficient than oligomers and large nanoparticles.

Bare PDAM nanoparticles are roughly spherical, and they can be somehow considered similar to the natural melanin granules obtained from hair or sepia ink, although obviously, they contain only decarboxylated units, and they lack small amounts of other biomolecules present in natural material. To avoid insolubilization of the PDAM particles and stabilization as the colloidal solutions, other components would be added to the media, such as polyvinyl alcohol. In the presence of this alcohol, the PDAM nanoparticles grow slowly, and oligomer intermediates were isolated and identified as cycled indole derivatives, including 2,7′- and 2,4′-cross-linked bonds [32,33]. Similar approaches have been used for the formation of water soluble PDAM, such as prior treatment of DHI with the hydrophilic triethylenglycol [60].

**Figure 4:**
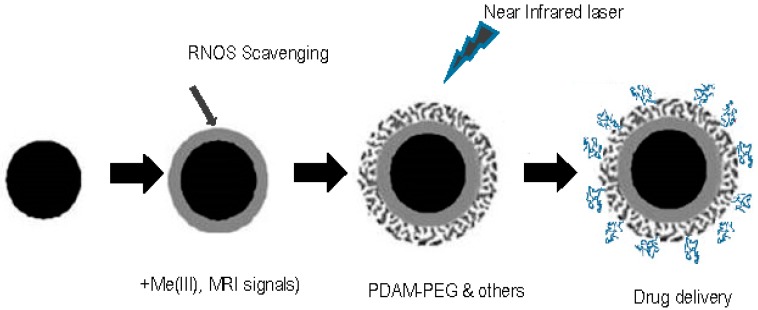
PDAM nanoparticle and biomedical applications. The PDAM nanoparticles can be directly used, coated, co-polymerized with ions, such a as Gd(III) or Fe(III) for MRI or conjugated with other materials on the surface (i.e., activated PEG). Alternatively, primary nanoparticles of other material can be coated with PDAM. These nanoparticles can be used for antioxidant therapy or for photothermal therapy after laser irradiation. Finally, they can be charged by non-covalent interactions with active antitumoral agents for drug delivery. The alternatives may be combined in different stages according to the final application.

PDAM coating is frequently applied to nanoparticles of diverse materials, or PDAM nanoparticles can be grafted with other materials for different functional uses. Thus, a second peripheral layer or PDAM co-assembly can be used for tuning the chemical properties of the surface. Sometimes it is convenient with an external PDAM-surface, and sometimes it is convenient with a PDAM-core conjugated with other material at the external surface, or with simultaneous co-assembly during DA polymerization (Figure 4).

PDAM coating of nanoparticles of different materials, such as glass, mica, graphene, silicon [60], or SiO_2_/silica [46,59], improves their electrical conductivity and optoelectronic properties. This treatment has been applied to some devices, such as superconductors [27], energy batteries [28,52], semiconductors [60], sensors [42,61], and biosensors [62,63]. DA have been also used for coating metal and metal oxide nanoparticles, such as titanium, tin dioxide, and indium/tin oxide glass [15,44], for experimental photostimulated batteries. In the case of SnO_2_ [55], the nanoparticles were submitted to a double treatment consisting of coating with PDAM, followed by carbonization of that surface. The resulting nanoparticles showed high electrical conductivity. Compared with the anode made of bare SnO_2_ nanoparticles, C–PDAM/SnO_2_ anode has significantly improved capacity and efficiency. On the other hand, the combination of PDAM with metal ions can be exploited to deposit a uniform metal covering preformed PDAM nanoparticles [38,63,64]. These materials have been also used for biological implants. Thus, Fe(III)-melanin nanoparticles are used as probes for MRI purposes. Magnetic tubular structures are also obtained by assembly of diphenylalanine with PDAM/magnetic Fe_3_O_4_ nanoparticles. These tubular hydrogel composites might be convenient as vehicles for tissue regeneration and drug delivery [65].

PDAM can also be conjugated with a variety of organic molecules forming co-polymers and composites. Activated PEG is one of the most frequently used molecules used in this line. Reactive functional groups are needed in PEG preactivation. Thiolated or aminated PEG facilitates covalent binding to PDAM for the formation of DPAM–PEG nanoparticles [10]. These nanoparticles show reduced cellular attachment to fibroblast in comparison to bare PDAM [38]. Similarly, activated DTPA has also be used. Furthermore, the PDAM–DTPA nanoparticles can be grafted with metals, such as gadolinium, to get structures with high efficiency, for diagnosis and therapy of cancer cells [66]. Conjugation of PDAM with hyaluronic acid has been used to increase cell adhesion and interactions with the extracellular matrix, and formation of mixed nanoparticles with gold [67]. PDAM has also been applied to modify clay and alumina. These modified materials are then used for incorporation to polypropylene [68] and polyphenylene sulfide polymers [69], to enhance their UV absorption and photostability.

Layer-by-layer PDAM nanoparticles can be synthesized by successive cycles of immersion in the appropriate solutions of DA and a second component. Thus, nanoparticles with around 30 alternative layers of PDAM and polyallylamine have been obtained by successive immersion, rinsing, and drying in DA and allylamine solutions. These nanoparticles show increased lifespan and improved optoelectronic properties than non-PDAM polyallylamine counterparts [70]. PDAM multilayers have also been recently prepared by the sequential deposition of DHI and polyphosphate using cerium as an oxidant for PDAM formation [71]. The oxidation of DHI by Ce(IV) takes place even at low pH, allowing a surface-controlled reaction, and the deposition of successive bi-component layers in acidic conditions.

Finally, sometimes quick and complete polymerization of precursors to form a melanin film is not required. l-dopa can be absorbed in zeolite nanoparticles without polymerization conditions. This composite material has been proposed for treatment of Parkinson disease as a way for slow delivery of dopa for Parkinson treatment [72].

## 2. Melanin in Biomedical Applications

### 2.1. Melanin in Magnetic Resonance Imaging (MRI)

The use of melanin in medical applications started a long time ago. In 1994, a patent was registered for the use of melanin as a contrast agent in magnetic resonance imaging (MRI) probes. The probe comprised of a combination of melanin with a signal-inducing metal exposed to emission, to improve detection of high-frequency soundwaves [73]. A mere suspension of melanin granules in a metal ion solution allows a stable charging of the melanin with the metal, due to its very high affinity to a number of transition and rare metal cations (dissociation constants around 10^−20^). These metals should be preferentially paramagnetic, or superparamagnetic, for MRI. Gd(III) was first proposed as the most promising for imaging enhancement [74]. According to the patent, metal ions emitting gamma rays might also be charged in melanin-based nanoparticles, but so far, this approach has never used in clinical diagnosis, due to the harmful side effects of gamma radiations. Melanin-PEG nanoparticles, complexed with Fe(III) ions, show higher relaxivity values than Gd(III) or Mn(II), with significant enhancement to MRI contrast [75]. To improve the signal, the optimal ion loading of these nanoparticles as therapeutic imaging probes has been recently calculated [73], as intra-nanoparticles’ antiferromagnetic coupling among proximal Fe(III) ions decreases the intensity of the signal.

### 2.2. Melanin in Antioxidant Therapy

PDAM nanoparticles have been frequently proposed as powerful antioxidants, to protect against damage by free radicals, ROS, and RONS (reactive oxygen and nitrogen species). The nanoparticles display good stability in water, and stronger free radical scavenging activity, to slow down the oxidation rate of soluble markers such as DPPH or ABTS [9]. In practice, PDAM has been proposed for treatment of severe diseases related to oxidative stress, such as neurologic disorders and inflammatory diseases. RONS, particularly hydroxyl radical and superoxide and peroxinitrite anions, are involved in the triggering of inflammation in ischemic strokes associated with myocardial infarction and brain injury [76]. PDAM–PEG nanoparticles are robust quenching agents of these species, but the levels of blood parameters, including aminotransferases, alkaline phosphatase, albumin, and urea, remain unaltered one day after injection, confirming excellent biocompatibility [7,10]. In addition, nanoparticles remain active in the blood during long periods, although some nitration is finally detected due to RONS scavenging. Recent studies have proven that PDAM–PEG nanoparticles behave as anti-inflammatory and neuroprotective agents in rat models [77]. Pre-injection of nanoparticles into rat brain lateral ventricles diminished RONS generation, and injection in blood remarkably decreased the levels of cytokine expression, making rats less vulnerable to ischemic stroke and inflammation. Recently, PDAM-coated hemoglobin nanoparticles have also been proposed to be effective hemoglobin-based oxygen carriers [78], as the PDAM coating inhibits the oxidation of hemoglobin to met-hemoglobin, but the hemoglobin maintains a high oxygen affinity.

In a different approach, injections of PDAM have been used as a radioprotector against radiation damage, particularly for protection of hematopoietic tissues that are very sensitive to irradiation. Spleen of mice pre-injected i.p. with 50 mg/kg of PDAM nanoparticles before exposure to 7 Gγ γ-irradiation dose, were clearly protected against control untreated mice [79].

Nevertheless, the antioxidant and anti-ROS activity of melanin should be always considered with caution, as it is dependent on the melanin conditions and aging. Natural melanin behaves as a double-edged sword, as it is basically a photoprotective intracellular antioxidant agent able to scavenge RNOS formed after UV radiation in skin melanocytes and retinal epithelial cells, but it is also a potentially harmful pro-oxidant agent under specific stress conditions [80,81]. A recent investigation has detected the generation of dangerous hydroxyl radicals from UV-irradiated PDAM [82]. This double face accounts for the use of melanin with opposite purposes to those exposed above. Sometimes endogenous melanin has been proposed as a putative target for chemotherapy of heavily pigmented melanotic melanoma, due to the pro-oxidant, rather than antioxidant melanin effect, once this endogenous pigment is highly exposed to oxygen and metal ions. These two agents induce a synergistic stress in malignant melanocytes, causing disorganization of melanosomes and high RONS generation, exceeding melanin capability scavenging, and consequently resulting in reduced viability of melanoma cells [83].

### 2.3. Melanin in Photothermal Therapy

Photothermal therapy is a relatively new and alternative technique to treat several types of solid cancer. It consists of the accumulation of an appropriate energy-absorbing agent in the localized tissue, to focus onto it, a near-infrared laser, causing therapeutical local hyperthermia, and the subsequent death of the surrounding malignant cells. 

In this regard, carbon and metal nanoparticles of different metals (Pt, Ce, Mn, and V) were first developed, but they were not well metabolized, and finally caused adverse health effects and toxicity, including altered mitochondria respiration, immunological response, and genotoxicity [84]. PDAM nanoparticles have been revealed as a convenient alternative to this photothermal treatment, due to their high energy-absorbing capacity after irradiation with laser. PDAM shows a high absorption efficiency in the infrared region, high conversion of that energy to heat, and remarkable stability to irradiation [10].

As mentioned above, PDAM nanoparticles are biocompatible [7,8,9,10] and well dispersed in blood, so they remain stable for several months, to repeat eventually new irradiations without new injections. Thus, it has been demonstrated that PDAM nanoparticles are able to kill tumor cells in animal models with short irradiation laser times [10]. This approach has been tested with HeLa and 4T1 cells. More than 90% of HeLa cells remained alive in the presence of the PDAM particles without laser irradiation, but less than 20% remained alive after irradiation for 5 min with 808 nm laser at 2 W/cm^2^ in the presence of 0.2 mg/mL DPAM. For solid tumors, DPAM nanoparticles were intratumorally injected into 4T1 solid tumors in Balb/c mice, and then irradiated with laser at the same doses. Most of the tumor tissue become necrotic, and no regrowth was observed during the next 10 days. After laser exposure in the absence of the DPAM, relevant regrowth occurred. These preliminary experiments in mice have shown that the toxic dose of colloidal PDAM was estimated around 0.5 mg/g body weight, 500 times higher than the dose needed for photothermal therapy, once the nanoparticles are located in the target tissue (normal concentration for injection is around 0.2 mg/mL). Nanoparticles are slowly metabolized by the action of endogenous oxidases, with relatively few adverse effects. Usually, human cells need around 1 h with a temperature rise to 42 °C for death, but in the presence of PDAM nanoparticles at the referred doses of laser irradiation, tumor tissue is easily heated to over 50 °C in around 5 min.

### 2.4. Melanin in Chemotherapy and Theranostics

Novel efficient treatments for diseases based on a single step combination of diagnostic and therapeutic capabilities, are named theranostics [85]. This approach requires the search for new bioinspired materials that combine the theranostic action with good biocompatibity, and relatively long half-life circulation, to avoid immune system recognition. As already stated, PDAM permits an easy coating of nanoparticles, a good capacity to be conjugated with other materials and composites, and good delivery of nanoparticle-charged active agents to reach specific targets.

According to this, the MRI, antioxidant, or photothermal therapies detailed above, have been improved using PDAM nanoparticles conjugated with other materials. These nanoparticles are tailored to be carriers of specific anticancer drugs that should be first preloaded, and later released in the target tissues in response to appropriate stimuli [7]. So far, that release has been triggered with laser infrared light (photothermal activation), but also by pH decrease [86]. PDAM–PEG nanoparticles have been loaded with anticancer drugs, such as doxorubicin and 7-ethyl-10-hydroxycamptothecin [86], or sorafenib [87]. The drug preloading in nanoparticles should involve non-covalent bonds for triggering the delivery by laser irradiation, or the acidic conditions usually occurring at the microenvironment of the tumor. The combination of the photothermal and the antibiotic cytotoxic effects can give place to a synergistic action on malignant cells, and is a very promising line of research (Figure 4).

On the other side, once again, it is worth mentioning that the endogenous melanin content of malignant melanoma can also be used for radiotherapy and chemotherapy with PDAM-free metal nanoparticles, due to the affinity of the endogenous pigment for those nanoparticles. For instance, Gadolinium-based nanoparticles formed by absorption of this metal in a polysiloxane network have been tested for theranostic treatment of melanoma, due to the affinity of the melanin contained in malignant melanocytes for these nanoparticles [66].

### 2.5. Melanin as a Complement for Tissue Scaffolds and Sealing Material

According to its adhesive and biocompatible properties, initial preliminary experiments showed that PDAM allows cell growth without toxic side effects on in vitro cultures. In contrast, cell culture flasks coated with thin films of PDAM stimulated the Schwann cell attachment, and in vitro neurite growth and extension of PC12 cells. The PDAM films improved the use of collagen [8]. In turn, PDAM has been used as conducting polymer for tissue scaffold. PDAM coating is very efficient for the improvement of biocompatibility in the construction of new materials in bone biosubstitutes, and in restoration of damaged or malfunctioning tissues [88]. Pre-deposition of a thin PDAM layer on organic polylactic–glicolic acid scaffolds by immersion of this material in a DA solution facilitates the posterior immobilization of osteoinductive molecules (BMP2, bone morphogenetic protein 2 and others) in stem cell-mediated bone tissue engineering, for bone regeneration and healing tissue defects [89].

On the other hand, in relation to the mfps-mimicking adhesive properties, PDAM has been recently conjugated with polyaspartamides, to form water-swollen polymer glues [90]. These glues show good adhesion with common materials, such as aluminum foil, glass, paper, and plastic. More importantly, in biomedicine, they have also demonstrated good adhesion when applied to biological skin. In addition, adhesive hydrogels by combination of PDAM–chitosan with thiolated pluronic have been proposed as a sealing and hemostatic antibleeding material [91]. Chitosan is a linear beta-polysaccharide formed by glucosamine and *N*-acetyl-glucosamine that is conjugated with DA for incorporation of catechol units to the glycosidic chain, mimicking mfps during the formation of the adhesion plaques in mussels (Figure 2). Pluronic is a polymer of ethylene and propylene oxides added to the hydrogel as antifoaming agent. The mixture of the three components, chitosan, PDAM and pluronic, gives place to a viscous hydrogel solution that rapidly undergoes an instantaneous solidification at the body temperature when injected in soft and mucous tissues [91].

### 2.6. Miscellaneous. Other Applications of PDAM

The exceptional features of PDAM allow its use in many other applications. For instance, synthesized PDAM nanoparticles have been proposed as “pseudonatural” photoprotectors in sunscreens. After addition of those nanoparticles to human epidermal keratinocytes, they are easily endocytosed to form a perinuclear cap that protect cell nuclei from UV damage, mimicking the behavior of natural melanosomes [92]. PDAM can also be combined with metal oxides, clays, and textiles, as photostabilizers and UV filters [68,69,93] of those materials, and assembled onto silica particles, to modulate the thickness of the wall in biodegradable drug-containing capsules [94]. This can be useful for increasing the time required for wall degradation, thus slowing drug delivery.

Aside biomedical applications, the hydroxy, carboxyl, and quinone groups found in melanin made this material a high affinity and high capacity agent to retain metal ions. This has been applied for water purification in metal-contaminating waste waters from chemical industries [95]. According to Langmuir curves, melanin extracted from human hair, or dopa-synthetic melanin deposited on discs of PVDF, show a high affinity for Pb(II), Cu(II), Cd(II), and Zn(II), in decreasing order. At the maximal binding capacity, melanin binds around 9% of lead (in mass). The technological applications of PDAM coating are extended to other related fields. Very recently, PDAM coating has been used for preparation of composite membranes applied to filtration and separation processes. Tailored PDAM coating on polyacrylonitrile substrate can form robust and long lifespan membranes, with uniform nanopores able to be used with good solvent permeability and high solute rejection. These membranes are appropriated for nanofiltration and molecular ultrafiltration of usual salts in aqueous solutions, as well as for recycling of metal catalyst nanoparticles from organic solvents [56]. In addition, related to the very sensitive detection of low levels of Pb(II) and Cu(II) in water, the synthesis of silver-melanin nanoparticles has been reported [96]. In this particular report, the melanin precursor was dopa instead of DA, possibly to increase the binding of the nanoparticles to metal ions, due to the carboxyl groups retained in the dopa-derived melanin polymer.

Finally, glassy carbon electrodes, prepared with graphene and PDAM, have also been successfully used as sensors, for simultaneous determination of guanine and adenine in HPLC detectors. These electrodes show good stability, reproducibility, selectivity, wide linear range, and higher sensitivity, for purine base detection, than compared to bare graphene electrodes [62]. They show good sensitivity in a pH range from 2 to 12, depending on the melanin precursor used for the melanin incorporation to the device. Thin films of melanin on indium tin oxide and gold substrates have also been used for very sensitive pH biosensors [61].

## 3. Concluding Remarks

It has been traditionally assumed that animals cannot utilize light energy as directly as plants, but eventually, it was proposed that melanin is a powerful molecule that goes far beyond being just a cutaneous photoprotective pigment in skin. Thus, it has been proposed, the possibility of direct light conversion for metabolism, minimizing mitochondrial energy production [97]. This “photomelano-metabolism” is complemented with theories contained in the book entitled “Melanin: the Human Chlorophyll”, that amazingly proposes melanin as a material for obtaining natural, cheap, and clean energy, by the generation of electrical energy from water with melanin-based autorenewable photoelectrochemical cells [98].

Although the proposals of Goodman and Solis-Herrera [97,98] should be considered exaggerated without further scientific evidence, advances on melanin applications suggest that this pigment, and the synthetic like-molecules, are indeed excellent materials for bioelectronics and biomedical purposes, due to their optoelectronic energy-related properties, metal binding capacity, adhesive properties, and their ability to form easy composites with other materials. The catecholic nature of melanin from animal skin, hair, and cuttlefish ink is shared with some adhesive proteins found in mussel foot. DA is a monomer that can easily form PDAM, a material sharing common properties with melanin (optoelectronic and energy-related properties), and mfps (adhesive properties for coating surfaces).

DPAM structure seems to depend on the polymerization conditions. It consists of uncycled DA, possibly cross-linked with cycled indole/indoline units. Other variants describe tetramer porphyrin-like indole units forming protomolecules in an onion-like structure, or just a DA polymer with non-covalent bonds. Composite PDAM nanoparticles can be obtained by conjugation of DPAM formed from DA solutions with a variety of organic and inorganic materials, or interactions with metal ions through the catechol groups. PDAM is easy and cheap to obtain, biocompatible, slowly biodegradable, tunable, and it does not induce cytotoxicity. Different variants of PDAM nanoparticles are being applied to engineering devices, including microcircuits of different conductivity, batteries, biosensors, and biomedical MRI, and photothermal therapy and drug delivery for cancer treatment. Melanin may serve as a material for improving sustainable technologies, and melanin research has illustrated an approach to explore further a combination of natural and synthetic like-materials. Intensive work is currently being performed, to design new melanin-related nanoparticles, for their application to multifunctional biomedical devices, as this offers an opportunity to combine diagnosis and therapy in theranostics.

## Abbreviations

H, K, P, Y	Amino acids letter code for Histidine, Lysine, Proline and Tyrosine
DA	Dopamine
Dopa	3,4-Dihydroxy phenylalanine
DHI	5,6-Dihydroxyindole
DHICA	5,6-Dihydroxyindole-2-carboxylic acid
DPTA	Diethylenetriaminepentacetate
Mfps	Mussel foot proteins
MRI	Magnetic Resonance Imaging
PDAM	Polydopamine Melanin
PEG	Polyethylenglycol
PTFE	Polytetrafluoroethylene
PVDF	Polyvinyledendifluoride
RNOS	Reactive Nitrogen Oxygen Species
Tris	Tris-(hydroxymethyl)aminomethane
